# Bay Leaf (*Laurus Nobilis* L.) Incense Improved Scopolamine-Induced Amnesic Rats by Restoring Cholinergic Dysfunction and Brain Antioxidant Status

**DOI:** 10.3390/antiox10020259

**Published:** 2021-02-08

**Authors:** Ion Brinza, Razvan Stefan Boiangiu, Monica Hancianu, Oana Cioanca, Ilkay Erdogan Orhan, Lucian Hritcu

**Affiliations:** 1 Department of Biology, Faculty of Biology, Alexandru Ioan Cuza University of Iasi, 700506 Iasi, Romania; ion.brinza@student.uaic.ro (I.B.); razvan.boiangiu@student.uaic.ro (R.S.B.); 2 Department of Pharmacognosy, Faculty of Pharmacy, Grigore T. Popa University of Medicine and Pharmacy Iasi, 700115 Iasi, Romania; monica.hancianu@umfiasi.ro (M.H.); oana.cioanca@umfiasi.ro (O.C.); 3 Department of Pharmacognosy, Faculty of Pharmacy, Gazi University, 06330 Ankara, Turkey; iorhan@gazi.edu.tr

**Keywords:** *Laurus nobilis*, bay leaf, incense, scopolamine, cholinergic system, memory, oxidative stress

## Abstract

Bay leaf (*Laurus nobilis* L.) has been shown to possesses various biological activities such as wound healing activity, antioxidant activity, antibacterial activity, antiviral activity, immunostimulant activity, anticholinergic activity, antifungal activity, insect repellant activity, anticonvulsant activity, antimutagenic activity, and analgesic and anti-inflammatory activity. The present study aimed to investigate whether the bay leaf incense (BL) elicits the memory formation via the action on the cholinergic system using a scopolamine (Sco)-induced rat model. Rats were exposed to BL over 5 min in a smoking chamber apparatus once daily for 22 days, whereas memory impairment was induced by Sco (0.7 mg/kg), a muscarinic receptor antagonist, delivered 30 min before each behavioral test. The phytochemical composition of BL was achieved by gas chromatograph–mass spectrometry (GCMS). Behavioral effects in rats were assessed by Y-maze, radial arm maze (RAM), and novel object recognition (NOR) paradigms. Additionally, the acetylcholinesterase (AChE) activity and the oxidative stress markers in the rat hippocampus were also evaluated. Exposure to BL significantly ameliorated Sco-induced cognitive impairment and oxidative stress in the rat hippocampus. The obtained results suggested that BL-induced ameliorative cognitive effects are mediated by enhancement of the cholinergic system and antioxidant activities.

## 1. Introduction

Cholinergic neurotransmission has been implicated in a variety of disease states [1]. In several types of dementia, including Alzheimer’s disease (AD), the cholinergic system is recognized as a significant factor because acetylcholine (ACh) plays an important role in cognitive processes [2]. A variety of cholinergic drugs has been approved in clinical trials to treat or enhance AD, exerting therapeutic effects by preventing ACh insufficiency and thereby increasing ACh levels in the brain, hydrolyzed by acetylcholinesterase (AChE) [3]. A meta-analysis of 26 studies of donepezil, rivastigmine, and galantamine reported a modest but clinically significant overall benefit of these drugs for cognition, function, behavior, and global clinical change stabilization [4]. The AChE inhibitors, such as donepezil, rivastigmine, and galantamine are the most common drugs prescribed for the treatment of AD because they temporarily increase ACh availability in cholinergic synapses [5]. The use of cholinergic medications for AD has limitations because they tended to cause adverse effects, such as pain, nausea, and hepatotoxicity [6,7]. Alternative treatment options are therefore required for AD patients.

Scopolamine (Sco) administration may be considered as a psychopharmacological model of AD [8]. Sco, an antagonist of the muscarinic receptor, inhibits the activity of the muscarinic acetylcholine receptor and the concomitant occurrence of temporary cognitive amnesia and electrophysiological changes close to those seen in AD [9,10]. Supporting evidence suggested that Sco-induced cognitive deficits via cAMP/SIRT1/Akt/Bcl-2 pathway [11] and also mediated brain oxidative stress [12]. Besides, growing evidence indicates that Sco interferes with extracellular signal-regulated kinase (ERK) and cAMP response element-binding (CREB)/brain-derived neurotrophic factor (BDNF) molecular homeostasis in animal models [13,14] while simulating ERK/CREB/BDNF injury in the brains of AD patients [15]. The Sco-induced model exhibited reduced ACh levels and choline acetyltransferase (ChAT) activity, with an increase in AChE activity in a previous study [16].

*Laurus nobilis* L. (Lauraceae) is a shrub that is native to the Mediterranean area and cultivated in a range of Asian countries, Europe as a spice or used as an ornamental plant, and the Americas [17,18]. Bay laurel (*L. nobilis* L.) leaves, sometimes referred to as laurel, noble laurel, sweet bay, or sweet laurel, have long been used for flavoring foods, particularly in Mediterranean cuisine as well as in cosmetology [19]. The leaves are traditionally used as a carminative, stomachic, and nervine [17,20], as well as in the treatment of amenorrhea, colic, condylomata, hysteria, polyps, sclerosis, and spasms [21]. In Iranian folk medicine, the leaves are used to treat epilepsy, neuralgia, and parkinsonism [21].

The chemical composition of the bay laurel leaves has been extensively studied. 1,8-Cineole (30.8%) has been identified to be the main compound followed by α-terpinyl acetate (14.9%), α-terpineol (8.0%), sabinene (7.9%), terpinen-4-ol (6.0%), α-pinene (5.3%), β-pinene (3.6%), methyleugenol (3.6%), and γ-terpinene (3.3%) [17]. Nabila et al. [22] reported 1,8-cineole (30.1%), α-terpynyl acetate (21.6%) and methyl eugenol (16.9%) as the main compounds in the chemical composition of the essential oil of *L. nobilis* leaves. It has been demonstrated that α-pinene and 1,8-cineole protected U373-MG cells against H_2_O_2_-induced oxidative injury by attenuating the loss of cell viability and cell morphology, inhibiting reactive oxygen species (ROS) production and lipid peroxidation, while increasing the endogenous antioxidant status [23]. Also, linalool and 1,8-cineole exhibited anticancer activities in lung cancer A549 cells [24]. Sabinene (8.86%), one of the major compounds from the needles of *Abies koreana*, contributed to the observed memory-enhancing effect on Sco-induce amnesia in mice [25]. Despite extensive studies about the biological activities of *L. nobilis* extract, there is currently no study that addressed the benefits of bay leaf incense (BL) in diseases involving brain disfunction, where the incense of the plant is sold with a memory-enhancing claim in Anatolia. Therefore, the present study was organized to elucidate the possible potential effects of BL on memory function and brain antioxidant status of rats exposed to Sco by means of scientific approach.

## 2. Materials and Methods 

### 2.1. Plant Material

The leaves of *L. nobilis* were provided in 2018 by Bakkalim Co, Ltd. (Mersin, Turkey), collected from its natural habitat in the Mediterranean region of Turkey.

### 2.2. Gas Chromatography-Mass Spectrometry (GC-MS) Analysis

Component analysis of the bay leaf smoke was performed using gas chromatography (Agilent 7890A, Agilent, Santa Clara, CA, USA)-mass detector (Agilent 5975C, Agilent, Santa Clara, CA, USA) device and equipped with a DB-5ms column (Agilent, Santa Clara, CA, USA, 30 m × 250 μm × 0.25 μm). The instrument control parameters included an injection of 0.25 µL with helium as the carrier gas, spitless mode, the oven program was 40 °C for 3 min, then increased 10 °C/min to 280 °C and maintained for 3 min, the flow was set to 1 mL/min, the purge flow to split vent was 40 mL/min at 1 min and the registered average velocity was 36.262 cm/sec. The total run time was 30 min and three injections were analyzed consecutively. The results represent the average value for the determinations. All identified compounds were calculated by comparison of the mass spectra to the standards and the literature data in the NIST/Wiley libraries as well as the data available in the Wiley 275 software from the used GC-MS system. The confirmation of each identified compound was verified against the linear retention indices (RI) with the published data in the scientific literature.

### 2.3. Animals

A total of 25 three-month-old male Wistar rats were used in the present study. The animals were housed in a temperature and light-controlled room (22 °C, a 12-h cycle starting at 08:00 h) with free access to food and water. All experimental procedures were strictly conducted by the Directive 2010/63/EU of the European Parliament and of the Council of 22 September 2010 on the protection of animals with approval from the Ethical Committee (No. 15309/22.07.2019).

### 2.4. Drug Treatment and Group Division

**Figure 1:**
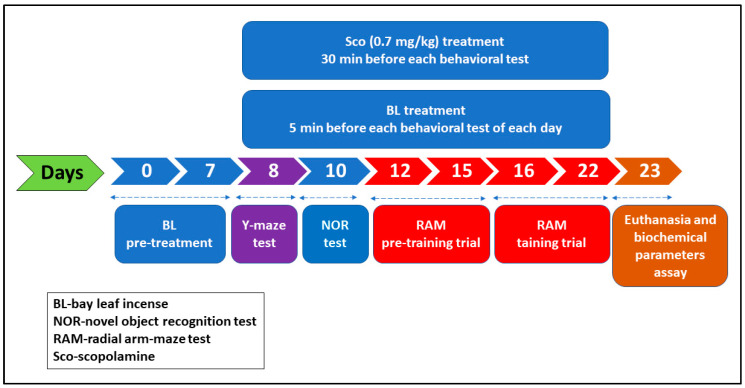
Schematic representation of drug treatment and behavioral analysis.

The scheme of drug treatments and behavioral measurements is summarized in Figure 1. Rats were randomly divided into five groups (*n* = 5 animals/group): first group—control; the second group—received donepezil treatment (DP, 5 mg/kg, Sigma-Aldrich, Darmstadt, Germany), as the positive control in the Y-maze, the novel object recognition and the radial arm-maze tests); the third group—received bay leaf incense (BL); the fourth group—scopolamine (Sco, 0.7 mg/kg, Sigma-Aldrich, Darmstadt, Germany) and fifth group—Sco received BL (Sco + BL). Also, we confirm that *n* = 5 animals/group is appropriate using InVivoStat, and R-based statistical package [26]. Based on a significance level of 0.05, the power to detect a 20% biologically relevant change from control is 90%. Rats were subjected to BL for 5 min during 7 days before starting behavioral tests and continuously administered during behavioral tests (22 days). The control and BL groups were intraperitoneally (i.p.) injected with 0.9% physiological saline. DP (5 mg/kg, Sigma-Aldrich, Darmstadt, Germany) and Sco (0.7 mg/kg, Sigma-Aldrich, Darmstadt, Germany) were dissolved in 0.9% physiological saline and i.p. injected, once daily, 30 min before the Y-maze, the novel object recognition and the radial arm-maze tests [27].

### 2.5. Exposure Chamber

Rats were exposed to BL following the method described by Merali et al. [28]. Before behavioral tests, rats were individually placed within the exposure chamber for 5 min. The exposure chamber was connected via rubber latex tubing to a vaporizing chamber containing a heating coil and was also connected to an electric pump, which created a gentle vacuum to pull the BL through to the exposure chamber. The animals in the control, DP, and Sco groups were subjected to similar conditions, except that the tube from the heating coil was disconnected to allow for the entry of fresh air. Separate exposure chambers and tubing were used for control and experimental groups. 

### 2.6. Behavioral Assessments 

#### 2.6.1. Y-Maze Spontaneous Alternation Test

Short-term spatial memory was assessed by recording spontaneous alternation behavior in a single-session Y-maze as previously described by Jackson [29] and Boiangiu et al. [27]. At 5 min after BL inhalation, each rat was placed at the end of one arm and allowed to explore the Y-maze freely for 8 min. The number of arm entries and sequence of arm visits were recorded using ANY-maze^®^ software (Stoelting CO, Wood Dale, IL, USA). Besides, the spontaneous alternation percentage was calculated as (number of alternation/total entries−2) × 100.

#### 2.6.2. Radial Arm-Maze

The radial arm-maze test (RAM) was used to explore working memory as well as reference memory for 5 min session after inhaling the BL as previously described by Olton and Samuelson [30] and Boiangiu et al. [27]. ANY-maze^®^ software (Stoelting CO, Wood Dale, IL, USA) was used to record the movements of rats inside the RAM. Before the training trial, there were four days of habituation sessions. During the training sessions (one trial per day for one week), the performance of individual rats was evaluated by the number of working memory errors (number of entries into baited arms visited before) and number of reference memory errors (number of entries into nonbaited arms). 

#### 2.6.3. Novel Object Recognition

The preference of novelty in rodents and the memory abilities were detected by a novel object recognition (NOR) task according to a method described previously by Foyet et al. [31]. The preference of a novel object may indicate the existence of familiar object presentation in the memory of a rat [32]. The animal behavior was analyzed by ANY-maze^®^ software (Stoelting CO, Wood Dale, IL, USA). The test equipment consisted of an open box (72 cm × 72 cm × 36 cm) in which rats were placed for two consecutive sessions (T1 and T2) with and an inter-trial interval of 1 h. The ”sample” trial (T1) comprising two identical objects, whereas during the ”choice” trial (T2), one familiar object (F) was replaced by a novel object (N), and the animal behavior was recorded for 5 min. T1 was performed at 5 min after BL inhalation. The discrimination index (DI) was calculated as the time spent in exploring N (TN)−the time spent in exploring F (TF)/TN + TF. Exploration was defined as sniffing or touching the object with the nose and/or forepaws. Sitting on the object was not considered exploration.

### 2.7. Measurement of the Biochemical Parameters

For biochemical assays, rats were deeply anesthetized (pentobarbital sodium, 150 mg/kg, b.w., i.p., Tokyo Chemical Industry, Tokyo, Japan), and decapitated for brain extraction. For homogenate preparation (1:10), hippocampi were centrifuged (15 min at 960× *g*, 4 °C) with ice-cold 0.1 M KH_2_PO_4_ buffer (pH 7.4) containing 1.15% KCl using a Potter Homogenizer (Cole-Parmer, Vernon Hills, IL, USA). The obtained supernatant was further assayed to investigate the AChE, superoxide dismutase (SOD), and catalase (CAT) specific activities, the total content of reduced glutathione (GSH), protein carbonyl, and malondialdehyde (MDA) levels.

#### 2.7.1. Determination of the AChE Activity

To measure the AChE (EC 3.1.1.7), the method of Ellman et al. [33] was used. The reaction mixture (600 μL) consisted of 0.26 M phosphate buffer with pH 7.4, 1 mM 5.5′-dithio-bis-2 nitrobenzoic acid (DTNB), and 5 mM acetylthiocholine chloride (ATC). The absorbance of the solution was read at 412 nm. The AChE activity was expressed as nmol of ACT/min/mg of protein. 

#### 2.7.2. Determination of the SOD Activity

Superoxide dismutase (SOD, EC 1.15.1.1) activity was determined using a procedure described by Winterbourn et al. [34]. The reaction mixture (1.5 mL) consisted of 100 mM TRIS/HCl (pH 7.8), 75 mM NBT, 2 μM riboflavin, 6 mM EDTA and 200 μL supernatant. The absorbance was measured at 560 nm. One unit of SOD activity was defined as the amount of enzyme causing 50% inhibition in the tetrazolium dye reduction rate. The enzyme activity was reported in U/mg protein.

#### 2.7.3. Determination of the CAT Activity

The catalase (CAT, EC 1.11.1.6) activity was measured based on the method described by Sinha [35]. The reaction mixture consisted of 150 μL phosphate buffer (0.01 M, pH 7.0) and 100 μL supernatant. The absorbance was read at 570 nm. The activity of the enzyme was expressed as μmol of H_2_O_2_ consumed/min/mg protein.

#### 2.7.4. Determination of the Protein Carbonyl Level

The method to determine the carbonylated protein levels, according to Oliver et al. [36] and modified through Luo and Wehr [37], is based on the reaction of 2,4-dinitrophenylhydrazine (DNPH) with the carbonyl groups of proteins. The results were expressed as nmol/mg protein.

#### 2.7.5. Determination of the MDA Level

To measure malondialdehyde (MDA) level, the method described by Ohkawa et al. [38] was employed. This method is based on the reaction of MDA, an aldehyde resulting from oxidative damage to cellular lipid membranes with thiobarbituric acid, resulting in a pink color, determined by a colorimetric method, measured in a spectrophotometer at 532 nm. The results were presented as nmol/mg protein.

#### 2.7.6. Estimation of the Protein Content

To determine the total protein, the method described by Smith et al. [39] was used. A bicinchoninic acid (BCA) protein assay kit (Sigma-Aldrich, Darmstadt, Germany) was used for the quantification of total protein. 

### 2.8. Statistical Analysis

All data were expressed as the mean ± standard error of the mean (S.E.M.). GraphPad Prism 7.0 software (La Jolla, CA, USA) was used to evaluate the obtained data. The data from different treatment groups were compared using one-way analysis of variance (ANOVA) followed by Tukey’s *post hoc* multiple comparison test. Differences were considered statistically significant at *p* < 0.05. Statistical correlations were expressed as Pearson correlation coefficient (*r*).

## 3. Results and Discussion

### 3.1. Phytochemical Analysis

**Figure 2:**
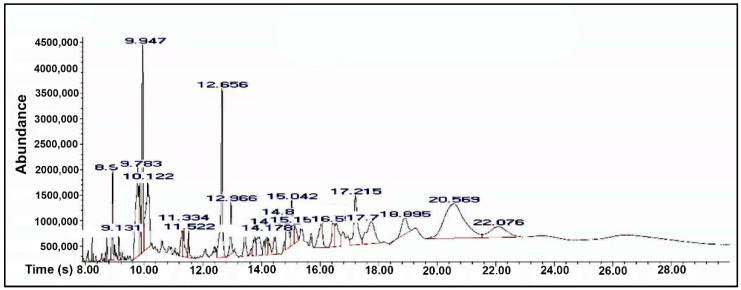
Gas chromatography-mass spectrometry (GC-MS) profile of the *L. nobilis* leaves smoke.

The bay leaf smoke was analyzed by GC-MS analysis (Figure 2), and its chemical composition was presented in Table 1. 37 compounds, corresponding to 97.29% of the total smoke were identified in the bay leaf smoke. Although the GC-MS analysis allowed us to identify only 97.29% of the total components present in the BL, the rest up to 100% (2.71%) included various substances as traces (each found in less than 0.2%) mainly represented by alkanes which we considered the pyrolysis artifacts and nonsignificant for our tests because their presence in the chromatogram is mainly in the second half. This indicates a higher mass lower volatility as compared to monoterpenes. However, their presence in the animal brain needs further testing and represents the basis of our future research. The most abundant chemical classes of the smoke components were oxygenated monoterpenes (44.84%), followed by oxygenated compounds (23.27%), sesquiterpenes (22.40%), and monoterpene hydrocarbons (6.78%). The major components of the bay leaf smoke were methyl dihydrojasmonate (14.72%), 1,8-cineole (12.61%), terpinen-4-ol (6.92%), trans-caryophyllene oxide (5.00%), carvacrol (4.76%), and citronellal (4.04%). This indicated that the obtained volatile compounds were from a 1,8-cineole chemotype laurel. However, significant amounts of sesquiterpenes and other types of oxygenated compounds were identified as well. All monoterpenes amounted to 50% of the volatiles, whereas sesquiterpenes were around half of the monoterpene quantity. Caryophyllene oxide, cubebene, and caryophyllene were the predominant sesquiterpenes. Nevertheless, the eight sesquiterpene compounds were all above 1%. Although most of the identified compounds possess fragrant, antimicrobial, repellant, anti-infective, and plant hormone properties, their combination, and ratio present a diversity of putative effects. The majority of the volatile substances found in lower concentrations in our sample may play an important role in enhancing the bioavailability of the main compounds. This is explained by the tensioactive properties of such volatiles in pharmaceutical technology essential oils being used as promoters for the absorption of active compounds [40]. Essential oils are complex mixtures of various small molecules substances and their chemical composition is highly influenced by environmental factors and processing technology. On the other hand, even if it uses the same raw material as an essential oil the incenses or smoke obtained by direct burning of the vegetal product comprises a different spectrum of compounds. This is mainly because the burning induces direct pyrolysis of the plant material and it takes a shorter amount of time than usual hydrodistillation. In our study we investigated the incense obtained during the first 5 min of burning, considering that this process leads to artifacts and metabolites that can suffocate the animals if inhaled for longer.

Today, little is known of the direct implications of the volatiles on the brain, especially the synergistic and multitarget approach of such compounds. Most of the research was directed on monoterpenes containing hydroxyl groups (such as linalool, eugenol, terpineol, terpinene-4-ol, etc.) and their synergistic effects with antibiotics enhancing the antimicrobial activity. However, the activity is correlated with the chirality, the size, the shape, and the physicochemical properties of the molecule and its interaction and integration in the cell membranes influencing the integrity/permeability, receptors, ionic channels, enzymatic systems, etc. [41,42,43]. Interestingly, the isomers of the volatile compounds can modify the odour, the taste, and the effects on the organisms. 

Previous data states that linalool and its derivatives have a great impact on the central nervous system (CNS) by interacting either with Ca^2+^ channels or by interfering with the muscarinic receptors, thus inducing anxiolytic and antidepressant effects. Similar effects and mechanisms have been postulated also for limonene, carvone, and O-methyl jasmonate. The lipophilic character of the compounds allows an easy passage through the blood-brain barrier. Such direct effects on the limbic systems have been proven for the first time for 1,8-cineole [44].

Our data agrees with many other authors working on *L. nobilis* essential oil. Dammak et al. [45] reported that 1,8-cineole was the most abundant compound of *L. nobilis* essential oil (43.2  ±  1.7%). Other main components of this essential oil were found to be β-terpinyl acetate (13.7  ±  1.9%), eugenol methyl ether (11.7  ±  1.8%), linalyl acetate (5.8  ±  0.6%), sabinene (4.1  ±  0.9%), α-pinene (4.1  ±  0.3%), as well as terpinen-4-ol (3.6  ±  0.3%) and β-pinene (3.2  ±  0.9%). Taban et al. [46] mentioned the presence of α-pinene (2.94–4.11%), β-pinene (2.51–3.22%), cis-sabinene hydrate (0.49–1.03%), eugenol (0.55–1.03%), methyleugenol (2.34–3.99%) and trans-caryophyllene (0–1.33%) in the chemical composition of the L. nobilis essential oil. Constituent analysis indicated the presence of volatile compounds such as isoeugenol (53.50%), myrcene (16.60%), and chavicol (10.20%) in the chemical composition of the L. nobilis essential oil [47]. In the analysis of the essential oil of L. nobilis leaves, Al-Kalaldeh et al. [48] reported 1,8-cineole (40.91%) as the major component, followed by α-pinene (5.82%), β-pinene (4.55%), sabinene (6.92%), limonene (2.10%), linalool (1.29%), and α-terpinyl acetate (5.86%). Upon these studies, we concluded that our BL has a chemical composition comparable to those reported by other groups and could support its cognitive-enhancer and antioxidant profile.

### 3.2. Effects of Bay Leaf Incense (BL) on Memory Formation in Behavioral Tasks

**Figure 3:**
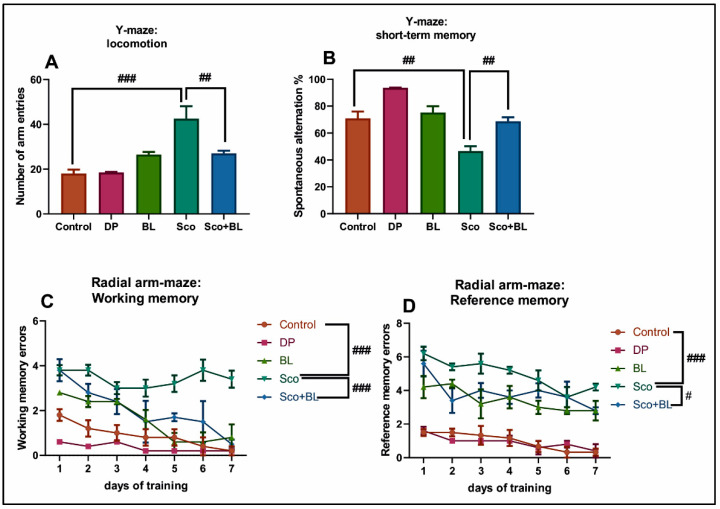
Behavioral analysis for the Y-maze and the radial-arm maze (RAM) tests. (**A**) represents the graph for the number of entries in the Y-maze test in different groups; (**B**) represents the graph for the spontaneous alternation percentage in the Y-maze test in different groups; (**C**) represents the graph for the working memory errors in RAM in different groups; (**D**) represents the graph for the reference memory errors in RAM in different groups. Data are expressed as means ± SEM (*n* = 5 and statistical analysis by one-way ANOVA followed by Tukey’s *post hoc* analyses: (**A**) Control vs. Sco: ### *p* < 0.0001, and Sco vs. Sco + BL: ## *p* < 0.001; (**B**) Control vs. Sco: ## *p* < 0.001, and Sco vs. Sco + BL: ## *p* < 0.001; (**C**) Control vs. Sco: ### *p* < 0.0001, and Sco vs. Sco + BL: ### *p* < 0.0001; (**D**) Control vs. Sco: ### *p* < 0.0001, and Sco vs. Sco + BL: # *p* < 0.01.

To explore whether BL exposure attenuated Sco-induced cognitive impairment in rats, the Y-maze, radial arm-maze (RAM), and novel object recognition (NOR) tests were conducted. For the Y-maze test, the Sco administered group demonstrated an increase of locomotor activity, as evidenced by a significant increase (*p* < 0.0001) of the number of arm entries compared to the control group (Figure 3A). When Sco was injected, a significant decrease of short-term memory performance was observed, as evidenced by decreased spontaneous alternation percentage (*p* < 0.001) compared with the control group (Figure 3B). Moreover, significant improvement of the spontaneous alternation percentage (*p* < 0.001) was observed in Sco-injected rat following BL exposure when compared to Sco-induced rats, indicating that the memory impairment induced by Sco was reversed. Compared with the control group, in the RAM, rats treated with Sco showed increased number of working memory errors (Figure 3C) (*p* < 0.0001), and number of reference memory errors (Figure 3D) (*p* < 0.0001). However, rats treated with Sco and BL showed significantly reduced number of working memory errors (Figure 3C) (*p* < 0.0001), and the number of reference memory errors (Figure 3D) (*p* < 0.01), when compared to the Sco-treated group. When BL and DP, a standard drug for AD, were administered, significant effects on memory performance in the Y-maze and RAM tests were noticed. 

**Figure 4:**
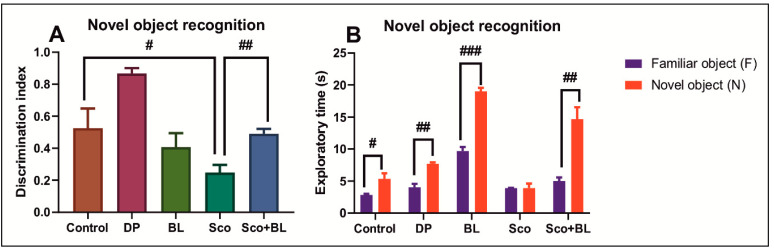
Behavioral analyses for the novel object recognition (NOR) test. (**A**) represents the graph for the discrimination index in different groups; (**B**) represents the graph for the exploratory time (s) in different groups. Data are means ± S.E.M. (*n* = 5) and statistical analysis by one-way ANOVA followed by Tukey’s *post hoc* analyses: (**A**) Control vs. Sco: # *p* < 0.01; Sco vs. Sco + BL: ## *p* < 0.001; (**B**) # *p* < 0.01; ## *p* < 0.001 and ### *p* < 0.0001.

Based on the outcomes, the Sco groups showed decreased discrimination index of the N (Figure 4A) (*p* < 0.01) as compared to the control group, while exhibited the same preference to explore both F and N, indicating an impaired response to recognizing the N. The Sco-treated rats subjected to BL, displayed significant increase (*p* < 0.001) in both discrimination index (Figure 4A) and the time to explore the N (Figure 4B), suggesting a positive response to novelty. Both BL and DP significantly increased performance in the NOR, suggesting positive effects on recognition memory. 

Few studies are describing the potential effects of *L. nobilis* against AD-relevant insults, consistent with our data obtained in the current study. People around the world have also used bay leaves in traditional and complementary medicine practices for hundreds of years. It has been demonstrated that bay leaf burning offers a range of health benefits. Anxiety relief is touted as a major benefit of bay leaf burning. This is probably because bay leaf smoke contains linalool, a compound found in several other plants. Pacifico et al. [49] demonstrated that *L. nobilis* leaf extracts have neuroprotective potential and anti-amyloidogenic efficacy. It has been reported that the chloroform fraction of *L. nobilis* was able to protect against cerebral ischemia neuronal damage [50]. Correspondingly, the apolar *L. nobilis* leaf extracts exhibited neuroprotective action toward three nervous system cell lines [51]. Moreover, some of the major compounds identified in the chemical composition of our *L. nobilis* used samples, supported its cognitive-enhancing profile. Lee et al. [52] demonstrated the neuroprotective potentials of α-pinene against Sco-induced learning and memory impairment in C57BL/6 mice. Moreover, the authors reported that α-pinene significantly increased the spontaneous alternation percentage in the Y-maze test, enhanced spatial recognition in the Morris water-maze test by reducing the escape latency, and increased step-through-latency in the passive avoidance test in the Sco-induced model. Recently, our group demonstrated that an essential oil mix containing β-pinene (1.76%), α-pinene (1.01%), linalool (0.55%), and cymene (0.53%) produced an improving effect on the consolidation of NOR memory [27]. Goto et al. [53] demonstrated a significant improvement of the cognitive function of elderly people following 1,8-cineole exposure. 1,8-cineole was previously proven to be a good anti-inflammatory and antinociceptive agent in mice models, whereas its isomer 1,4-cineole demonstrated good anxiolytic effects [54]. Interestingly, the main oxygenated compound identified in our sample, methyl dihydrojasmonate (syn. hedione) promotes neuronal health as indicated by Pavan et al. [55]. Moreover, this compound proved to be efficient in neurodegenerative diseases associated with pigmentation problems due to its binding to the vomeronasal type-1 receptor 1 (VN1R1) found in the amygdala and hippocampus. Furthermore, olivetol is recognized as a diphenol cannabinoid compound with antioxidant and anticholinergic properties [56]. In line with these results, our in vivo findings confirm the ability of the BL to usefully modulate and enhance cholinergic neuronal transmission and cognitive performance under dementia-related conditions. 

### 3.3. Effects of Bay Leaf Incense (BL) on AChE-Inhibiting Activity

**Figure 5:**
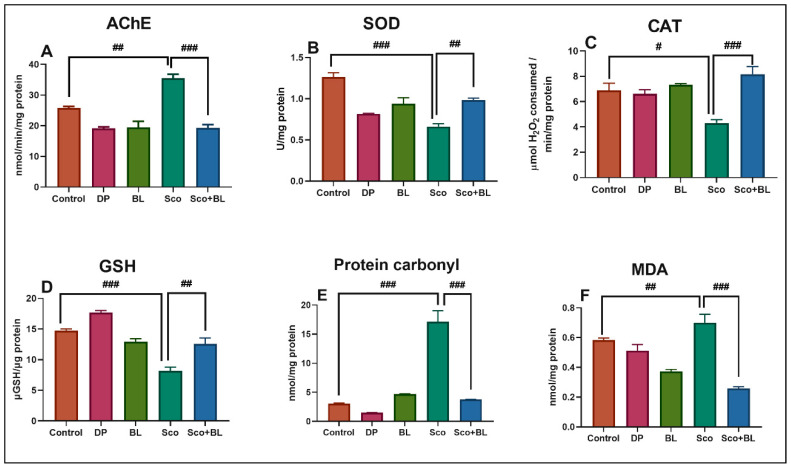
Effects of the bay leaf incense (BL) on (**A**) AChE; (**B**) superoxide dismutase (SOD); (**C**) glutathione peroxidase (GPX) specific activities; (**D**) reduced glutathione (GSH); (**E**) protein carbonyl and (**F**) malondialdehyde (MDA) level. Values represent means ± S.E.M. (*n* = 5) followed by Tukey’s *post hoc* analyses: (**A**) Control vs. Sco: ## *p* < 0.001, Sco vs. Sco + BL: ### *p* < 0.0001; (**B**) Control vs. Sco: ### *p* < 0.0001, Sco vs. Sco + BL: ## *p* < 0.001; (**C**) Control vs. Sco: # *p* < 0.01, Sco vs. Sco + BL: ### *p* < 0.0001; (**D**) Control vs. Sco: ### *p* < 0.0001, Sco vs. Sco + BL: ## *p* < 0.001; (**E**) Control vs. Sco: ### *p* < 0.0001, Sco vs. Sco + BL: ### *p* < 0.0001; (**F**) Control vs. Sco: ## *p* < 0.001, Sco vs. Sco + BL: ### *p* < 0.0001.

In memory and cognitive functions, the cholinergic transmission plays a significant role. The enzyme AChE is responsible for the degradation of ACh into acetate and choline and decreases neurotransmitter levels in the brain, as can be noticed in the cholinergic dysfunction of AD [57]. Acetylcholinesterase inhibitors (AChEIs) increase the amount of ACh, improving memory functions. People with AD are commonly treated with procognitive medicines. However, the hepatotoxicity and the side effects arising from the activation of the cholinergic system limit the use of AChEIs [58,59]. The effect of BL on the AChE activity in the rat hippocampus caused by Sco is shown in Figure 5A. The activity of AChE significantly increased (*p* < 0.001) in the Sco-treated group as compared to the control group. However, the activity of AChE in the administration of the BL significantly decreased (*p* <0.0001) compared to the Sco-treated group. DP and BL exhibited anti-AChE activities. Our results demonstrated the high anti-AChE activity of the BL. These outcomes are in line with few past investigations which reported the anti-AChE activity of the *Laurus* extract. Gazwi et al. [60] demonstrated that *Laurus* leaf extract significantly restored AChE of the brain in lead-treated rats, proposing that *Laurus* extract could preserve living organisms against neurotoxicity by reversing the AChE imbalance caused by lead. This anti-AChE activity of the extract could be attributed to its phenolic and flavonoids contents and its antioxidant activity. Ferreira et al. [61] showed that *L. nobilis* extract exhibited high AChE inhibitory activity due to the presence of flavonoids. Our data suggest that the memory enhancement effects of BL in Sco-induced amnesic rats could be attributed to the inhibition of the AChE activity and restored of the cholinergic system activity. 

### 3.4. Effects of Bay Leaf Incense (BL) on the Hippocampus Oxidative Status

The condition of oxidative stress may be used to assess the hippocampus state of Sco-treated rats [62]. Sco exhibited pro-oxidant activity, as evidenced by suppressed activity of SOD (*p* < 0.001) (Figure 5B), CAT (*p* < 0.01) (Figure 5C), the total content of reduced GSH (*p* < 0.0001) (Figure 5D), along with increased levels of protein carbonyl (*p* < 0.0001) (Figure 5E), and MDA (*p* < 0.001) (Figure 5F). As an antioxidant agent, BL exposure restored the antioxidant enzyme activity and decreased the levels of protein carbonyl and lipid peroxidation as compared to Sco-treated animals.

Oxidative stress is one of the pathways responsible for Sco-induced amnesia. The pro-oxidative effects of Sco have been documented as it decreases the activity of antioxidant enzymes such as SOD, CAT, and GPX [63,64] and increased the concentration of malondialdehyde (MDA), which is the main marker of lipids peroxidation [64,65]. Also, numerous studies have shown the pro-cognitive impact of antioxidant compounds on Sco-induced memory damage, possibly by attenuating oxidative stress markers [27,58]. As demonstrated by a substantial increase in SOD and CAT specific activities and the total content of reduced GSH, along with a decrease in protein carbonyl and MDA levels, BL significantly restored the antioxidant status in the brain of rats. The current results are supported by different studies that demonstrated the antioxidant effects of *Laurus* extract. Hanaa et al. [60] indicated the antioxidant activity of *Laurus* leaves extract and assumed that it has a defensive role against the oxidative damage caused by lead in a rat’s brain. Turkez et al. [66] demonstrated the preventive role of *L. nobilis* leaf extract in alleviating aluminum phosphide-induced DNA and oxidative damages *in vitro*. Ham et al. [67] reported that spirafolide from the bay leaf (*L. nobilis*) prevented dopamine-induced apoptosis by decreasing reactive oxygen species production in human neuroblastoma SH-SY5Y cells. BL exposure effectively restored the antioxidant defense mechanism by increasing the antioxidant levels of activity in the brain.

**Figure 6:**
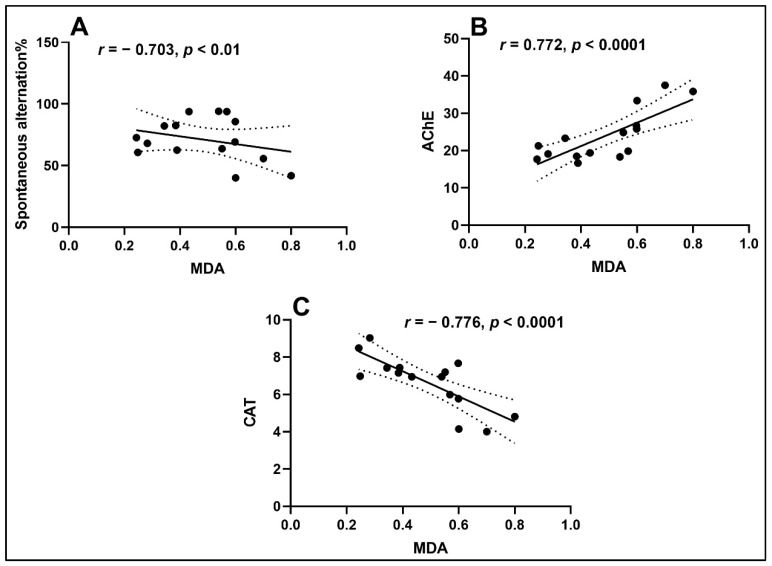
Statistical Pearson’s correlation analyses of behavioral scores and oxidative stress markers (*n* = 5). Values expressed are spontaneous alternation%, AChE (nmol/min/mg protein), CAT (U/mg protein), and MDA (nmol/mg protein). (**A**) Spontaneous alternation% vs. MDA (*n* = 5, *r* = −0.703, *p* < 0.01); (**B**) AChE vs. MDA (*n* = 5, *r* = 0.772, *p* < 0.0001); (**C**) CAT vs. MDA (*n* = 5, *r* = −0.776, *p* < 0.0001).

To determine the potential association between memory, antioxidant enzymes and lipid peroxidation, the Pearson correlation analysis was used (Figure 6). In this way, a significant negative correlation between the spontaneous alternation% vs. MDA (*n* = 5, *r* =−0.703, *p* < 0.01) (Figure 6A) and CAT vs. MDA (*n* = 5, *r* = − 0.776, *p* < 0.0001) (Figure 6C) was noticed, suggesting that memory improvement in the Y-maze test is well corelated with a low level of MDA. Also, a decreased activity of AChE is positive correlated with a low level of MDA (*n* = 5, *r* = 0.772, *p* < 0.0001) (Figure 6B). A correlation between the AChE inhibitory action of the ethanolic extract of *Laurus* leaf and its antioxidants ability in rats was shown by Gazwi et al. [60]. By using Pearson’s test, the improvement of spatial memory in behavioral approaches is significantly corelated with the decrease of AChE activity and lipid peroxidation level following BL exposure. 

## 4. Conclusions

In short, BL exposure may improve memory formation. At the same time, it can effectively diminish the cognitive deficits induced by Sco in the rat brain. The mechanism of the observed effects can be explained by decreasing in the AChE activity followed by increased level of antioxidant enzymes changed as a consequence of Sco administration. These new findings provide pharmacological and biochemical support for the development of the potential of BL in cognitive deficits.

## Figures and Tables

**Table 1:** The chemical composition (%) of *L. nobilis* L. leaf smoke.

No.	Compound	RI	Composition (%)
1.	*α*-Thujene	930	0.51
2.	*α*-Pinene	939	0.29
3.	Sabinene	977	0.52
4.	L-*β*-Pinene	982	0.14
5.	Myrcene	991	1.58
6.	*p*-Cymene	1025	0.70
7.	L-Limonene	1028	1.94
8.	*α*-Fenchene	1060	0.28
9.	*α*-Terpinene	1078	0.82
***Monoterpene hydrocarbons***			**6.78**
10.	Linalool	1103	0.87
11.	Orange/Rose oil	1124	2.25
12.	*cis*-Limonene oxide	1138	1.13
13.	Pinocarvone	1163	0.56
14.	Citronellal	1170	4.04
15.	1,8-Cineole	1183	12.61
16.	L-(-)-Menthol	1185	1.11
17.	Terpinen-4-ol	1187	6.92
18.	L-*α*-Terpineol	1189	0.64
19.	(R)-(+)-beta-Citronellol	1237	1.11
20.	D(+)-Carvone	1246	1.70
21.	Geraniol	1256	0.70
22.	*p*-Anisaldehyde	1258	0.24
23.	Cinnamic aldehyde	1267	1.48
24.	Carvacrol	1298	4.76
25.	Piperonal	1300	2.46
26.	Eugenol	1329	2.26
***Oxygenated monoterpenes***			**44.84**
27.	*trans*-Caryophyllene	1418	3.75
28.	Cyclamen aldehyde	1434	1.38
29.	*α*-Amorphene	1452	2.08
30.	Seychellene	1464	1.97
31.	*α*-Cubebene	1469	4.25
32.	*α*-Curcumene	1477	1.02
33.	*trans*-Caryophyllene oxide	15.81	5.00
34.	E-*cis*-*β*-Santalol	1616	2.95
***Sesquiterpenes***			**22.40**
35.	* Acetyleugenol	1525	3.71
36.	Olivetol	1755	4.84
37.	Methyl dihydrojasmonate	2276	14.72
***Oxygenated compounds***			**23.27**
**Total**			**97.29**

* placed in this position because it is not a sesquiterpene.

## Data Availability

The data presented in this study are available on request from the corresponding author.
